# Comparison of Analytical Values after Changing to the International Standardized Method for Lactate Dehydrogenase and Alkaline Phosphatase Measurements in Mouse and Rat

**DOI:** 10.3390/vetsci9110595

**Published:** 2022-10-27

**Authors:** Kayo Furumoto, Noboru Fujitani, Masakatsu Nohara, Akihisa Hata

**Affiliations:** 1 Faculty of Veterinary Medicine, Okayama University of Science, Ikoino-oka 1-3, Imabari 7948555, Japan; 2 Biomedical Science Examination and Research Center, Okayama University of Science, Ikoino-oka 1-3, Imabari 7948555, Japan

**Keywords:** lactate dehydrogenase, alkaline phosphatase, International Federation of Clinical Chemistry and Laboratory Medicine, isozyme, Japan Society of Clinical Chemistry, mouse, rat

## Abstract

**Simple Summary:**

Since April 2020 in Japan, lactate dehydrogenase and alkaline phosphatase assays, which were earlier conducted using the Japan Society of Clinical Chemistry protocols, are being conducted using the International Federation of Clinical Chemistry and Laboratory Medicine protocols. The correlation between blood values measured by these methods is not known in some species; therefore, values measured using these two methods cannot be used interchangeably. In this study, the relationship between lactate dehydrogenase and alkaline phosphatase values of mice and rats using each method was determined, and coefficients were generated to convert and compare the obtained values. These coefficients can be used for the mutual conversion of measured values during the transition period from the Japan Society of Clinical Chemistry method to the International Federation of Clinical Chemistry and Laboratory Medicine method. However, it should be noted that the conversion coefficients were affected by isozyme composition.

**Abstract:**

Since April 2020, the method for lactate dehydrogenase (LD) and alkaline phosphatase (ALP) activity measurements in Japan has been switched from the Japan Society of Clinical Chemistry (JSCC) reference method, which is only used in Japan, to the International Federation of Clinical Chemistry and Laboratory Medicine (IFCC) reference method. However, in some species, the relationship between the blood values of both enzymes measured by the two methods remains unclear. Hence, values measured by these two methods cannot be used interchangeably. Therefore, this relationship was examined in ICR mice and Wistar/ST rats. The LD and ALP values obtained by both methods were plotted on scatter graphs, and regression equations were obtained. To compare the JSCC (x) and IFCC (y) methods, regression equations were generated for LD values in non-hemolytic samples as follows: y = 0.954x − 4.008 for ICR mice and y = 0.963x − 6.324 for Wistar/ST rats. The conversion factors from the JSCC to the IFCC methods were 0.954 (mice) and 0.963 (rats). The conversion coefficients from the IFCC to the JSCC methods were 1.048 (mice) and 1.088 (rats). For ALP values in fasted mouse and rat samples, the regression equations were y = 0.336x − 2.247 and y = 0.314x − 17.626, respectively. The conversion factors from the JSCC to the IFCC methods were 0.336 (mice) and 0.314 (rats). The conversion coefficients from the IFCC to the JSCC methods were 2.978 (mice) and 3.188 (rats). These conversion factors can be used for the mutual conversion of both measured values during the transition period from the JSCC to the IFCC method. However, it should be noted that the conversion coefficients for both LD and ALP were affected by isozyme composition.

## 1. Introduction

Lactate dehydrogenase (LD; EC 1.1.1.27) is widely distributed in organisms and catalyzes the interconversion of lactic acid and pyruvate [1,2,3]. In human and veterinary medicine, blood LD activity is used as a marker of cell damage: generally, high LD activity indicates cellular abnormality [4,5]. There are five isozymes of LD, and their tissue distribution patterns vary among animal species [6,7,8,9]. Alkaline phosphatase (ALP; EC 3.1.3.1) is also ubiquitous from bacteria to humans and catalyzes the hydrolysis of phosphomonoesters at alkaline pH [10,11,12]. In humans, at least four isoenzymes encoded by the ALP gene have been identified: three are tissue-specific, namely intestinal, placental, and germ cell ALP, and one is tissue-nonspecific, including bone, liver, and kidney ALP [13]. Bone and some intestinal ALP have been reported in mouse blood [14,15], and liver, bone, and intestinal ALP have been reported in rat blood [16].

In laboratory animal research, blood biochemistry examinations, such as those of LD and ALP, are performed for various purposes. These examinations are commonly conducted using clinical examination devices, reagents, and methods designed for the evaluation of human samples. In Japan, the Japan Society of Clinical Chemistry (JSCC) reference method is used for LD and ALP measurements, while, internationally, the International Federation of Clinical Chemistry and Laboratory Medicine (IFCC) reference method is used. However, since April 2020, clinical laboratory institutions in Japan have switched their LD and ALP measurement methods to those of the IFCC. It is thus unclear whether the supply of reagents for measuring LD and ALP activity through the JSCC method will continue in the future. Furthermore, the composition and pH of the buffer solution differ between the JSCC and IFCC methods, leading to different reactivities. When measuring LD activity, the JSCC method uses diethanolamine buffer (pH 8.8 at 30 °C) [17], while the IFCC method uses N-methyl-D-glucamine buffer (pH 9.4 at 37 °C) [18]; when measuring ALP activity, 2-ethylaminoethanol buffer (pH 9.90 at 30 °C) is used in the JSCC method [19], whereas 2-amino-2-methyl-1-propanol buffer (pH 10.20 at 37 °C) is used in the IFCC method [20]. Hata et al. [21] reported the relationship between ALP values in bovine, canine, and feline blood measured by these methods, but this relationship remains unclear in other animal species. Hence, the values measured using these two methods cannot be used interchangeably. Therefore, this study aimed to clarify the relationship between the JSCC and IFCC methods in terms of LD and APL values in mice and rats. Regression equations for LD and ALP values obtained by each method were analyzed using blood from mice and rats. The relationship between JSCC and IFCC measurements of LD and ALP is influenced by isozyme composition in humans [22]. Therefore, in this study, we evaluated the effect of hemolysis for LD and feeding for ALP—among the factors causing isozyme composition variation—on the relationship between the JSCC and IFCC methods. When both JSCC and IFCC values are available, these regression equations may be useful for the effective reuse of data and may have the effect of reducing the number of animals used, i.e., the “reduction” in the principle of the 3Rs (Replacement, Reduction and Refinement), in animal experiments.

## 2. Materials and Methods

### 2.1. Sample Collection

Forty-one mouse serum and 35 rat serum samples were used in this study. Of these samples, 36 samples were from fed mice (Slc:ICR: 10 males and 11 females, 8–37 week-old) and rats (Slc:Wistar/ST: eight males and seven females, 7–10 week-old) (Table 1). These animals were obtained from Japan SLC, Inc. (Shizuoka, Japan) and kept in cages in the laboratory animal center of our university. The laboratory animal center was kept at a temperature of 24–26 °C, humidity of 40–60%, and 12 h/12 h light/dark cycle. Animals were fed a pelleted diet (CE-2; CLEA Japan, Inc., Tokyo, Japan) and water ad libitum. The serum of the fed animals was separated from the residual blood from the training procedure using laboratory animals at our university. Sodium pentobarbital 50 mg/kg + medetomidine hydrochloride 0.75 mg/kg was administered intraperitoneally, and after confirming the disappearance of the pedal reflex, the skin and muscle were incised, and blood was drawn from the posterior vena cava. After blood collection, the abdominal aorta and posterior vena cava were severed, and cardiac and respiratory arrest in animals was confirmed. The blood was centrifuged at 2150× *g* at room temperature for 10 min. Mouse (300–400 μL) and rat (800–1200 μL) sera were collected in a cryovial and frozen at −80 °C until analysis. The Animal Care and Use Committee of Okayama University of Science approved this training procedure (approval number: 2020-087).

Forty serum samples of fasted mice (Slc:ICR: 10 males and 10 females, each 10 week-old) and rats (Slc:Wistar/ST: 10 males and 10 females, each 10 week-old) were obtained from Japan SLC, Inc. The animals were fasted overnight (16 h). Under anesthesia (2–3% isoflurane), mouse blood was drawn from the posterior vena cava, and rat blood was drawn from the abdominal aorta. After blood collection, the abdominal aorta and posterior vena cava were severed, and cardiac and respiratory arrest in animals was confirmed. These procedures were performed in compliance with the law and guidelines on animal experiments in Japan and the regulations of laboratory animal welfare in Japan SLC, Inc.

### 2.2. Analysis of Serum Lactate Dehydrogenase and Alkaline Phosphatase Activity

The LD and ALP activities were measured using JSCC and IFCC methods. Unless otherwise specified, reagents were obtained from Fujifilm Wako Pure Chemical (Osaka, Japan). The JSCC method for the LD analysis employed LD-J, which consists of L-lactate in diethanolamine buffer solution and β-nicotinamide adenine dinucleotide solution. The lower limit of quantitation was 5.4 U/L, and the upper limit was 1300 U/L. The IFCC method for the LD measurement employed LD-IF, which consists of L-lithium lactate in N-methyl-D-glucamine buffer solution and β-nicotinamide adenine dinucleotide solution. The lower limit of quantitation was 5 U/L, and the upper limit was 1300 U/L. For the analysis of LD and ALP, approximately 100 μL serum was processed in the automated analyzer. The actual serum volumes used for ALP analysis were 3 and 4 μL in the JSCC and IFCC methods, respectively, and 4 and 4 μL in the JSCC and IFCC methods, respectively, for LD analysis.

The ALP analysis was performed in the same manner as described in our previous study [21]. The JSCC method for the ALP analysis employed ALP II-J2, which consists of a 2-ethylaminoethanol buffer solution and 4-nitrophenyl phosphate-substrate solution. The lower limit of quantitation was 1.5 U/L, and the upper limit was 2000 U/L. The IFCC method for the ALP analysis employed ALP IFCC, which consists of a 2-amino-2-methyl-1-propanol buffer solution and 4-nitrophenyl phosphate substrate solution. The lower limit of quantitation was 1 U/L, and the upper limit was 700 U/L.

A Hitachi 3100 clinical analyzer (Hitachi High-Technologies Corp., Tokyo, Japan) was used for LD and ALP analyses. The enzyme calibrator Wako was used for the calibration. Control Wako-I and Wako-II were used for quality control.

As calculated using the JSCC method, the assigned LD values of control Wako-I and control Wako-II were 152 and 345 U/L, respectively. As calculated using the IFCC method, the assigned LD value of control Wako-I was 154 U/L and that of control Wako-II was 356 U/L. As calculated using the JSCC method, the assigned ALP values of control Wako-I and control Wako-II were 194 and 561 U/L, respectively. As calculated using the IFCC method, the assigned ALP value of control Wako-I was 70 U/L and that of control Wako-II was 229 U/L.

In both the JSCC and IFCC methods for LD analysis, the intra-assay coefficients of variation (CVs) using control Wako-I were 0.45% and 0.74%, respectively. The inter-assay CVs using control Wako-I were 0.60% and 0.99%, respectively. In the ALP analysis, the intra-assay CVs using control Wako-I were 0.74% and 0.85%, respectively. The inter-assay CVs using control Wako-I were 1.12% and 1.55%, respectively.

### 2.3. Analysis of Alkaline Phosphatase Isozyme

Agarose-gel electrophoresis was performed using an ALP isozyme analysis kit (Quick ALP (QG), Helena, Saitama, Japan) according to the manufacturer’s instructions. Thirty microliters of serum were used for each electrophoresis lane. The ALP isozyme bands were detected using 3-indoxyl phosphate disodium salt as the substrate and nitro blue tetrazolium as the dye. Intestinal ALP in serum was identified by inhibiting liver ALP and bone ALP with levamisole (levamisole hydrochloride; Nacalai Tesque, Kyoto, Japan) [23].

### 2.4. Statistical Analysis

The LD and ALP values obtained using both methods were plotted in a scatter graph. In the graph, the x-axis represents the values obtained by the JSCC method, and the y-axis represents those obtained by the IFCC method. The normality of the measurements for the fed and fasting groups and for the non-hemolyzed and hemolyzed samples was confirmed by the Kolmogorov–Smirnov test using IBM SPSS Statistics for Windows, version 19 (IBM Corp., Armonk, NY, USA). The probability of significance was set at 0.05. As the test demonstrated normal distribution in all groups, a parametric regression analysis was performed. The regression equation was obtained using the major axis regression method in Validation-Support/Excel Ver. 3.5 (JSCC, Quality Management Expert Committee, Tokyo, Japan). The 95% confidence intervals (CIs) were calculated using the bootstrap method. Differences in regression coefficients of the regression lines obtained from non-hemolyzed samples and hemolyzed samples, from fed samples, and from fasted samples were determined by testing the t-value.

The residuals of each regression formula were calculated as:

residual = (actual measurement value by the IFCC method) − (IFCC value estimated by regression formula)(1)

The standardized residuals of each regression formula were calculated as:

standardized residual = (residual)/(standard deviation)(2)

In addition, we used the slope of the regression equation of the standard principal axis regression analysis method as the conversion factor for LD and ALP measurement values.

## 3. Results

### 3.1. LD Activity in Mouse Samples and Regression Analyses between JSCC and IFCC Methods

**Figure 1:**
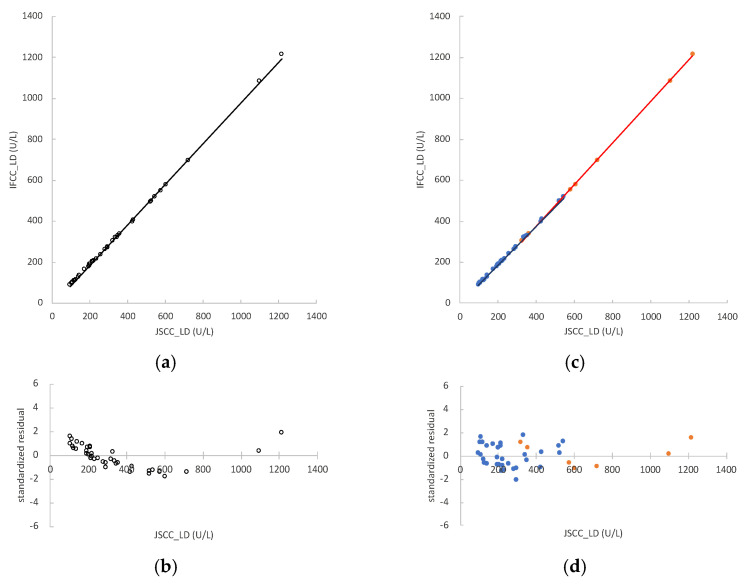
Correlation between lactate dehydrogenase (LD) activity measured by the Japan Society of Clinical Chemistry (JSCC) and International Federation of Clinical Chemistry and Laboratory Medicine (IFCC) methods in mouse serum samples. The x-axes represent values obtained by the JSCC method and the y-axes represent those obtained by the IFCC method: (**a**) regression analysis comparing the JSCC and IFCC values of all samples (*n* = 39), represented by the formula y = 0.990x − 12.956; (**b**) standardized residual plots obtained using the regression equation of all samples (*n* = 39); (**c**) regression analysis comparing JSCC and IFCC values of non-hemolyzed samples (*n* = 32, blue dots) and hemolyzed samples (*n* = 7, orange dots), represented by the formulas y = 0.954x − 4.008 and y = 1.015x − 31.033, respectively; (**d**) standardized residual plots obtained using the regression equations of non-hemolyzed samples (*n* = 32, blue dots) and hemolyzed samples (*n* = 7, orange dots).

In mouse serum samples (*n* = 41), the LD values measured by the JSCC and IFCC methods were 96–1662 U/L and 88–1654 U/L, respectively. Two samples were excluded from the regression analysis for deviating from the measurement range of the LD analytical reagent (>1300 U/L). The regression formula of the LD values of mouse serum was y = 0.990x − 12.956, and the correlation coefficient (r) was 0.9989 (Figure 1a). The 95% CIs of the regression coefficient and constant term were 0.959 to 1.002 and −16.350 to −4.597, respectively. The standardized residual plots for values < 600 U/L showed a tendency to decline for LD activity (Figure 1b), indicating that the fitting of the regression line was not appropriate.

To determine the cause of this heteroscedasticity, the samples were divided into two groups: non-hemolyzed and hemolyzed samples. In the non-hemolyzed samples (*n* = 32), the LD values measured by the JSCC and IFCC methods were 96–543 U/L and 88–518 U/L, respectively. The regression formula of the non-hemolyzed samples was y = 0.954x − 4.008, and r = 0.9980 (Figure 1c). The 95% CIs of the regression coefficient and constant term were 0.944 to 0.960 and −5.776 to −1.607, respectively. In the hemolyzed samples (*n* = 7), the LD values measured by the JSCC and IFCC methods were 323–1218 U/L and 305–1213 U/L, respectively. The regression formula of the hemolyzed samples was y = 1.015x − 31.033, and r = 1.0000 (Figure 1c). The 95%CIs of the regression coefficient and constant term were 0.967 to 1.037 and −50.372 to −5.045, respectively. The value “1” was not included in the regression coefficient for the 95% CI for non-hemolyzed samples. Therefore, there was a proportional systematic error. The constant term did not include the value “0” for the 95% CIs of the regression equations for non-hemolyzed and hemolyzed samples. Therefore, there was a certain constant systematic error. The regression coefficient of the hemolyzed sample regression line was significantly higher than that of the non-hemolyzed samples (*p <* 0.001). The residual values of the samples were homoscedastic (Figure 1d).

### 3.2. LD Activity in Rat Samples and Regression Analyses between JSCC and IFCC Methods

**Figure 2:**
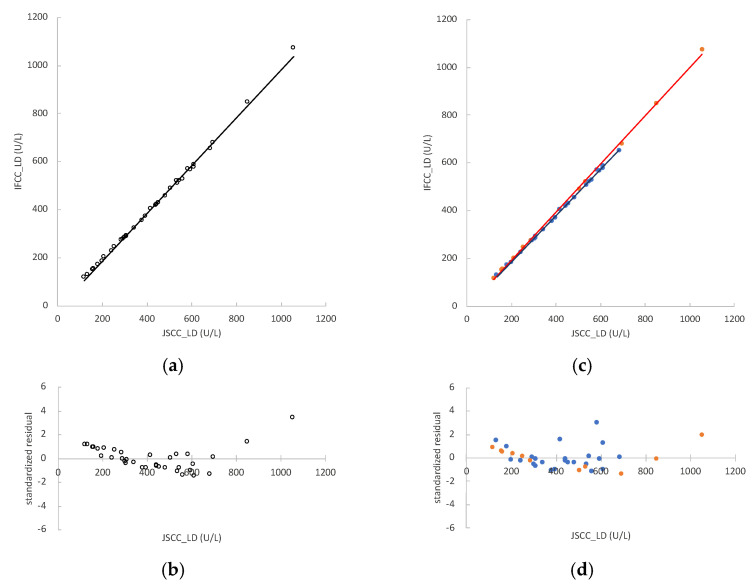
Correlation between lactate dehydrogenase (LD) activities measured by the Japan Society of Clinical Chemistry (JSCC) and International Federation of Clinical Chemistry and Laboratory Medicine (IFCC) methods in rat serum samples. The x-axes represent the values obtained by the JSCC method and the y-axes represent those obtained by the IFCC method: (**a**) regression analysis comparing the JSCC and IFCC values of all samples (*n* = 35), represented by the formula y = 0.997x − 16.252; (**b**) standardized residual plots obtained using the regression equation of all samples (*n* = 35); (**c**) regression analysis comparing the JSCC and IFCC values of non-hemolyzed samples (*n* = 24, blue dots) and hemolyzed samples (*n* = 11, orange dots), represented by the formulas y = 0.963x − 6.324 and y = 1.012x − 13.078, respectively; (**d**) standardized residual plots obtained using the regression equations of non-hemolyzed samples (*n* = 24, blue dots) and hemolyzed samples (*n* = 11, orange dots).

The LD values measured by the JSCC and IFCC methods in rat serum samples (*n* = 35) were 121–1056 U/L and 117–1073 U/L, respectively. The regression formula was y = 0.997x − 16.252, and r = 0.9992 (Figure 2a). The 95%CIs of the regression coefficient and constant term were 0.956 to 1.020 and −27.715 to −2.467, respectively. As with the residual plots of mouse values, the plots of standardized residual values were heteroscedastic (Figure 2b).

As mentioned above, the samples were divided into non-hemolyzed and hemolyzed samples. In the non-hemolyzed samples (*n* = 24), the LD values measured by the JSCC and IFCC methods were 134–684 U/L and 129–652 U/L, respectively. The regression formula of the non-hemolyzed samples was y = 0.963x − 6.324, and r = 0.9983 (Figure 2c). The 95%CsI of the regression coefficient and constant term were 0.951 to 0.978 and −11.670 to −1.295, respectively. In the hemolyzed samples (n = 11), the LD values measured by the JSCC and IFCC methods were 121–1056 U/L and 117–1073 U/L, respectively. The regression formula of the hemolyzed samples was y = 1.012x − 13.078, and r = 1.0000 (Figure 2c). The 95% CIs of the regression coefficient and constant term were 0.974 to 1.028 and −22.355 to −2.258, respectively. The value “1” was not included in the regression coefficient for the 95% CI for non-hemolyzed samples. Therefore, there was a proportional systematic error. The constant term did not include the value “0” for the 95% CIs of the regression equations for non-hemolyzed and hemolyzed samples. The regression coefficient of the hemolyzed sample regression line was significantly higher than that of the non-hemolyzed samples (*p* < 0.001). The heteroscedasticity of the residue plots in Figure 2d was improved compared to that in Figure 2b.

### 3.3. ALP Activity in Mouse Samples and Regression Analyses between the JSCC and IFCC Methods

**Figure 3:**
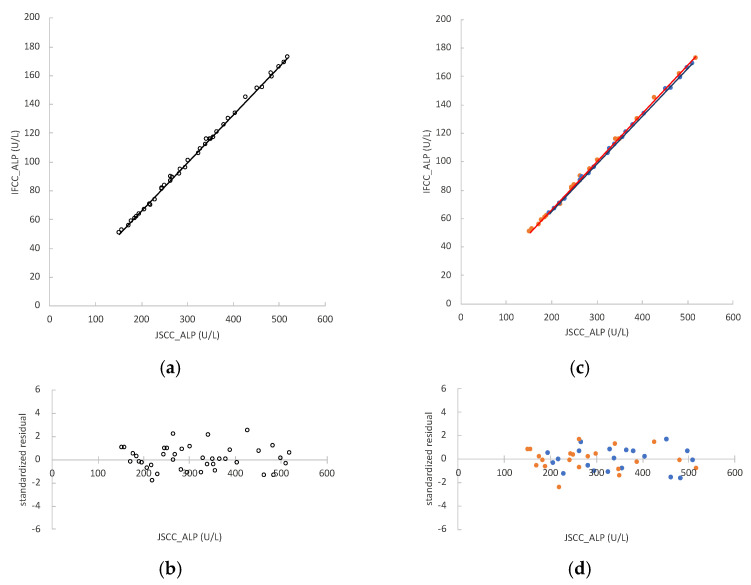
Correlation between alkaline phosphatase (ALP) activities measured by the Japan Society of Clinical Chemistry (JSCC) and International Federation of Clinical Chemistry and Laboratory Medicine (IFCC) methods in mouse serum samples. The x-axes represent the values obtained by the JSCC method, and the y-axes represent those obtained by the IFCC method: (**a**) regression analysis comparing JSCC and IFCC values of all samples (*n* = 41), represented by the formula y = 0.335x − 1.274; (**b**) standardized residual plots obtained using the regression equation of all samples (*n* = 41); (**c**) regression analysis comparing JSCC and IFCC values of fed mice (*n* = 21, orange dots) and fasted mice (*n* = 20, blue dots), represented by the formulas y = 0.339x − 1.515 and y = 0.336x − 2.247, respectively; (**d**) standardized residual plots obtained using the regression equations of fed mice (*n* = 21, orange dots) and fasted mice (*n* = 20, blue dots).

The ALP values measured by JSCC and IFCC methods in mouse serum samples (*n* = 41) were 152–518 U/L and 51–173 U/L, respectively. The regression formula was y = 0.335x − 1.274, and r = 0.9989 (Figure 3a). The 95% CIs of the regression coefficient and constant term were 0.332 to 0.338 and −2.475 to −0.182, respectively. The residual values of the samples were homoscedastic (Figure 3b).

The samples were further divided into two groups: fed and fasted mice. In fed mice (*n* = 21), the ALP values measured by the JSCC and IFCC methods were 172–518 U/L and 56–173 U/L, respectively. The regression formula of samples from fed mice was y = 0.339x − 1.515 and r = 0.9965 (Figure 3c). The 95% CIs of the regression coefficient and constant term were 0.335 to 0.344 and −3.101 to −0.171, respectively. In fasted mice (*n* = 20), the ALP values measured by the JSCC and IFCC methods were 196–510 U/L and 64–169 U/L, respectively. The regression formula of samples from fasted mice was y = 0.336x − 2.247 and r = 1.0000 (Figure 3c). The 95% CIs of the regression coefficient and constant term were 0.332 to 0.340 and −3.691 to −0.728, respectively. The value “1” was not included in the regression coefficient for the 95% CI for fed and fasted mouse samples. Therefore, there was a proportional systematic error, which is natural as the measured value by the IFCC method differs widely from that of the JSCC method. The constant term did not include the value “0” for the 95% Cis of the regression equations for either sample. Therefore, there is a certain constant systematic error. No statistically significant difference was observed in the regression coefficient between the fed mouse regression line and that of the fasted mice (*p* = 0.411).

### 3.4. ALP Activity in Rat Samples and Regression Analyses between JSCC and IFCC Methods

**Figure 4:**
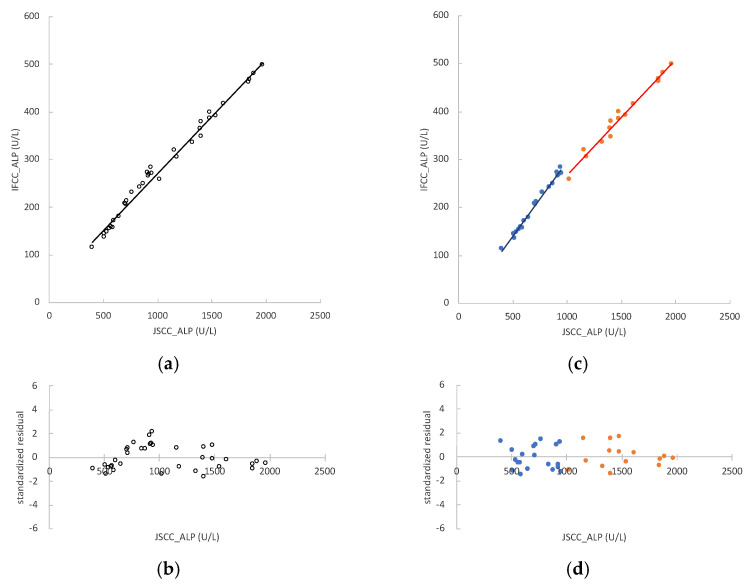
Correlation between alkaline phosphatase (ALP) activities measured by the Japan Society of Clinical Chemistry (JSCC) and International Federation of Clinical Chemistry and Laboratory Medicine (IFCC) methods in rat serum samples. The x-axes represent the values obtained by the JSCC method, and the y-axes represent those obtained by the IFCC method: (**a**) regression analysis comparing the JSCC and IFCC values of all samples (*n* = 35), represented by the formula y = 0.241x + 30.666; (**b**) standardized residual plots obtained using the regression equation of all samples (*n* = 35); (**c**) regression analysis comparing the JSCC and IFCC values of fed rats (*n* = 15, orange dots) and fasted rats (*n* = 20, blue dots), represented by the formulas y = 0.243x + 22.355 and y = 0.314x − 17.626, respectively; (**d**) standardized residual plots obtained using the regression equations of fed rats (*n* = 15, orange dots) and fasted rats (*n* = 20, blue dots).

The ALP values measured by the JSCC and IFCC methods in rat serum samples (*n* = 35) were 398–1967 U/L and 115–499 U/L, respectively. The regression formula was y = 0.241x + 30.666, and r = 0.9918 (Figure 4a). The 95% CIs of the regression coefficient and constant term were 0.232 to 0.250 and 20.353 to 41.361, respectively. The residual values of the samples were heteroscedastic (Figure 4b).

To identify the cause of this heteroscedasticity, samples were divided into two groups: fed and fasted rats. In fed rats (*n* = 15), the ALP values measured by the JSCC and IFCC methods were 1021–1967 U/L and 259–499 U/L, respectively. The regression formula of the samples from fed rats was y = 0.243x + 22.355, and r = 0.9876 (Figure 4c). The 95% CIs of the regression coefficient and constant term were 0.244 to 0.261 and −1.923 to 53.554, respectively. In fasted rats (*n* = 20), the ALP values measured by the JSCC and IFCC methods were 398–945 U/L and 115–284 U/L, respectively. The regression formula of the samples from fasted rats was y = 0.314x − 17.626, and r = 0.9835 (Figure 4c). The 95% CIs of the regression coefficient and constant term were 0.297 to 0.332 and −29.416 to −6.588, respectively. The value “1” was not included in the regression coefficient for the 95% CIs for fed and fasted samples. Therefore, there was a proportional systematic error. As earlier indicated, the proportional system error between JSCC and IFCC values is natural. The constant term did not include the value “0” for the 95% CIs of the regression equations for fasted samples. Therefore, there is a certain constant systematic error. The regression coefficient of the fasted rat regression line was significantly higher than that of fed rats (*p* < 0.001). The residual values of the samples were homoscedastic (Figure 4d).

### 3.5. Analysis of ALP Isozyme in Mouse and Rat Serum

**Figure 5:**
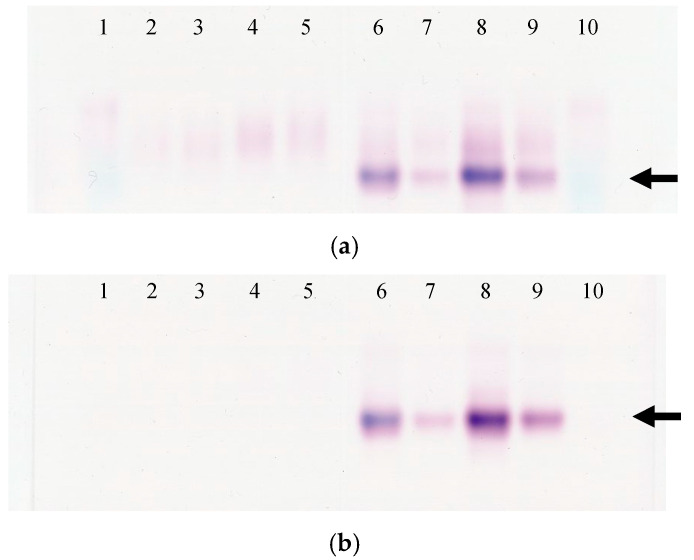
Electrophoretic patterns of alkaline phosphatase (ALP) isozyme in rat serum. (**a**) Gel not treated with levamisole. 1. control serum (human serum), 2. fed mouse serum (serum ALP 152 U/L analyzed by the JSCC method), 3. fasted mouse serum (207 U/L), 4. fed mouse serum (483 U/L), 5. fasted mouse serum (463 U/L), 6. fed rat serum (1021 U/L), 7. fasted rat serum (398 U/L), 8. fed rat serum (1401 U/L), 9. fasted rat serum (945 U/L), 10. control serum (human serum). (**b**) Gel treated with levamisole. 1. control serum (human serum), 2. fed mouse serum (serum ALP 152 U/L analyzed by the JSCC method), 3. fasted mouse serum (207 U/L), 4. fed mouse serum (483 U/L), 5. fasted mouse serum (463 U/L), 6. fed rat serum (1021 U/L), 7. fasted rat serum (398 U/L), 8. fed rat serum (1401 U/L), 9. fasted rat serum (945 U/L), 10. control serum (human serum). The arrow indicates the intestinal ALP band.

Mouse and rat serum samples were analyzed by electrophoresis to detect ALP isozyme bands. In levamisole-treated gels, the band disappeared in the mouse samples and remained in the rat samples. This remaining band corresponded to the intestinal ALP isozyme. In addition, the staining intensity of the intestinal ALP bands varied between the fed and fasted rat samples: intestinal ALP was more abundant in fed rat samples than in fasted rat samples (Figure 5).

### 3.6. Conversion Factor between JSCC and IFCC Methods for Measuring LD Values

**Figure 6:**
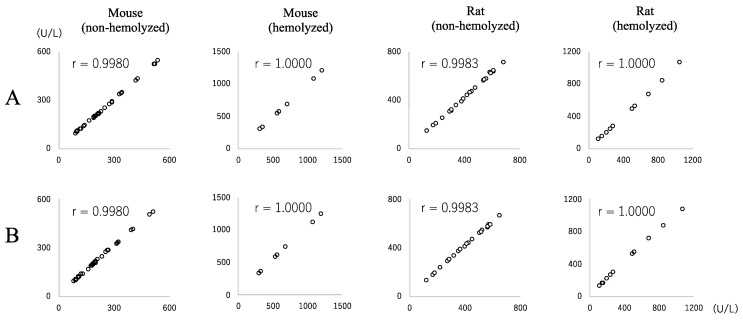
Scatter plots of the measured LD values (x-axis) and estimated LD values (y-axis) using the conversion factors. (**A**): Measured IFCC LD values (x) and estimated IFCC LD values (y) obtained from the JSCC values using the conversion factors. (**B**): Measured JSCC LD values (x) and estimated JSCC LD values (y) obtained from the IFCC values using the conversion factors.

When the regression coefficient was used as the conversion factor, the conversion factors from the JSCC to the IFCC methods for LD values were as follows: mouse (non-hemolyzed samples) 0.954, mouse (hemolyzed samples) 1.015, rat (non-hemolyzed samples) 0.963, and rat (hemolyzed samples) 1.012. The regression equation was generated by the standard major axis regression method with the IFCC and JSCC measurement values as x and y, respectively. The conversion coefficients from the IFCC to JSCC methods were as follows: mouse (non-hemolyzed samples) 1.048, mouse (hemolyzed samples) 0.985, rat (non-hemolyzed samples) 1.088, and rat (hemolyzed samples) 0.988. Figure 6. shows scatter plots of the measured values (x) and estimated values (y) using the conversion factors. In all plots, there were high correlations.

### 3.7. Conversion Factor between JSCC and IFCC Methods for Measuring ALP Values

**Figure 7:**
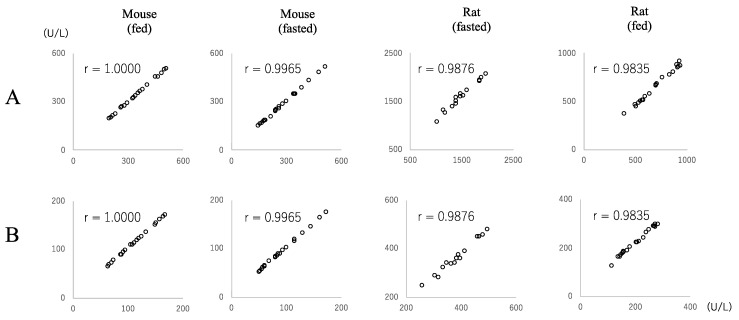
Scatter plots of the measured ALP values (x-axis) and estimated ALP values (y-axis) using the conversion factors. (**A**): Measured IFCC ALP values (x) and estimated IFCC ALP values (y) obtained from the JSCC values using the conversion factors. (**B**): Measured JSCC ALP values (x) and estimated JSCC ALP values (y) obtained from the IFCC values using the conversion factors.

When the regression coefficient was used as the conversion factor, the conversion factors from the JSCC to the IFCC methods for ALP values were as follows: mouse (fasted) 0.336, mouse (fed) 0.339, rat (fasted) 0.314, and rat (fed) 0.243. The regression equation was calculated by the standard major axis regression method with the IFCC and JSCC measurement values as x and y, respectively. The conversion coefficients from the IFCC to the JSCC methods were as follows: mouse (fasted) 2.978, mouse (fed) 2.952, rat (fasted) 3.188, and rat (fed) 4.107. Figure 7 shows scatter plots of the measured values (x) and estimated values (y) using the conversion factors. In all plots, there were high correlations.

## 4. Discussion

There are five types of LD isozymes, LD1, LD2, LD3, LD4, and LD5 [6,7,8,9], which differ in reactivity to reagents between the JSCC and IFCC methods. LD1 and LD5 have the most dissimilar subunit configurations; therefore, their reactivity with reagents is different [17,22]. The reactivity of human LD isozymes with reagents has been previously reported; while in the JSCC method, the measured LD5 value tended to be higher than the LD1 value, in the IFCC method, no significant differences between the values were found. Therefore, in a sample with a high content ratio of LD5, the LD value obtained by the IFCC method tends to be lower than that obtained by the JSCC method [17,22]. The other samples showed a good correlation between the two methods, with no significant difference in the values. The slope of the regression equation obtained through the JSCC and IFCC methods in healthy humans was 0.96; therefore, values from both methods can be treated almost equally if they are near the Japanese LD reference range [22]. LD5 is the predominant fraction in mouse and rat serum, liver, and skeletal muscle [8,24,25,26]. However, in this study, the regression equations of both methods for LD activity in non-hemolytic samples of mice and rats had the same slope as those of humans. Although the reactivity of each LD isozyme with reagents has not been clarified in mice and rats, the reactivity of LD measurement reagents with each LD isozyme may differ depending on the animal species. In addition, in this study, most of the samples with high LD values were hemolytic because there was a marked increase in serum LD due to hemolysis, as erythrocytes contain approximately 200 times more LD than serum. Additionally, the slope of the regression equation of hemolytic samples was significantly different from that of non-hemolytic samples. The difference in inclination between hemolytic and non-hemolytic samples may reflect the change in serum isozyme composition due to hemolysis.

In humans, bovine, canines, and felines blood, ALP values measured by the IFCC method were approximately one-third of those measured by the JSCC method [21,22]; this was caused by differences in the buffer solution compositions. Similar results were obtained for the mouse and rat samples in this study. Four types of ALP isozymes (tissue non-specific ALP isozymes present in bones, liver, and the kidneys; intestinal ALP isozymes; placental ALP isozymes; and germ cell ALP isozymes) have been reported in humans [13,27]. The reactivity between human ALP isozymes and reagents varies depending on the isozyme type. In particular, placental and intestinal ALP isozymes differ from other isozymes in their reactivity to reagents [19,22]; therefore, samples containing a high concentration of these types deviate from the regression equations obtained for the JSCC and IFCC methods. That is, the relationship changes in samples with increased placental ALP (typical in pregnant individuals) and intestinal ALP (common when samples are collected after meals or from individuals with blood type B or O).

In this study, the regression equations of the JSCC and IFCC methods used to measure ALP activity in samples of fasted mice and rats had the same slopes as those in humans. The regression equation slope of the fed mouse samples was similar to that of the fasted mouse samples, but the regression equation slope of the fed rat samples was significantly different from that of the fasted rat samples. Intestinal ALP was higher in fed rats, and the regression equation slope of the fed rats was smaller than that of the fasted rats, which is similar to the observation in humans [28]. The effect of feeding on blood ALP activity differs between mice and rats [29]: serum ALP activity of fed rats was higher than that of fasted rats [29,30,31], due to intestinal ALP [30,32]. Human intestinal ALP is known to be highly responsive to the JSCC method but not the IFCC method [19], meaning that samples containing large quantities of intestinal ALP present lower values when measured by the IFCC method. Although the reactivity of each rat ALP isozyme with these reagents has not been clarified, the difference in the regression equation slope between the fasted and fed rat samples may reflect the reactivity differences of both methods. In mice, fasting did not affect the slope of regression equations. Furthermore, intestinal ALP could not be detected by agarose gel electrophoresis in mouse serum. Previous studies have shown that small intestinal ALP in mouse blood was largely absent, and total blood ALP activity was not significantly different between fasted and fed mice [14,15]. This result supports the hypothesis that intestinal isozymes influence the slope of the regression equation in rats. However, since the serum ALP isozyme composition of mice and rats may differ depending on the strain, it is necessary to check the changes in the analytical values for the strain used in the experiment.

The correlation between the JSCC and IFCC methods for measuring blood LD and ALP values in mice and rats was similar to that of humans. The conversion factor in this study can be used for the mutual conversion of both measured values during the transition period from the JSCC to the IFCC methods. This may contribute to a reduction in the number of animals used from the perspective of animal welfare. However, it should be noted that, as in humans, the slope of the regression equation is affected by changes in isozyme composition.

The present study had some limitations. When performing human clinical laboratory testing, 50 or more patient samples are generally evaluated in order to conduct performance comparisons between assays. This is partly because larger inter-individual differences often exist when performing human studies, and larger sample numbers allow for more valid regression analysis. In the present study, blood samples from animals from a closed colony of the same animal supply company were used, so it was assumed that there would be fewer inter-individual differences. Thus, we judged that a smaller number of samples would not be significantly problematic. We believe that this is a reasonable solution for reducing the number of animals used. However, sera samples from one representative strain of both mice and rats were used. Furthermore, sera collected from drug-treated animals and animal models of disease were not included. Therefore, it is unclear whether the tentative regression equations obtained in this study can be used for all sample types. Furthermore, this study used frozen sera, and it is possible that degradation or LD and ALP activity changes may have occurred during frozen storage. In addition, the anesthetics used and the site of blood collection differ in feeding and fasting animals. Although there are no reports that the anesthetics or blood collection sites used in this study had a significant effect on serum LDH or ALP activity levels, this possibility cannot be excluded. Finally, the reagents used in the JSCC and IFCC methods were purchased from Fujifilm (Wako Pure Chemical Industries, Ltd.), and although both reagents are standardized, there may be some differences in reactivity compared to reagents from other companies.

## 5. Conclusions

In comparing the JSCC (x) and IFCC (y) methods, the regression equations for LD values in non-hemolytic samples were y = 0.954x − 4.008 in mice and y = 0.963x − 6.324 in rats. The conversion factors from the JSCC to the IFCC methods were 0.954 for mice and 0.963 for rats. The conversion coefficients from the IFCC to the JSCC methods were 1.048 for mice and 1.088 for rats. For the ALP values in fasted mice and rat samples, the regression equations were y = 0.336x − 2.247 and y = 0.314x − 17.626, respectively. The conversion factors from the JSCC to the IFCC methods were 0.336 for mice and 0.314 for rats. The conversion coefficients from the IFCC to the JSCC methods were 2.978 for mice and 3.188 for rats. These conversion factors can be used for the mutual conversion of both measured values during the transition period from the JSCC to the IFCC methods. However, it should be noted that the conversion coefficients for both LD and ALP were affected by isozyme composition.

## Figures and Tables

**Table 1:** Number of animals included in serum lactate dehydrogenase and alkaline phosphatase activity analysis.

Sample		Fed Animals	Fasted Animals	Total
Mouse	Male	10	10	20
	Female	11	10	21
Rat	Male	8	10	18
	Female	7	10	17
	Total	36	40	76

## Data Availability

The datasets generated and/or analyzed during the current study are available in the Open Science Framework, at https://osf.io/yw46k/?view_only=03eaa742acfe422fa3c08a731cd5bc79 (accessed on 25 October 2022).
